# Towards a more integrative environmental assessment: Infauna as tool for *Zostera marina* conservation management

**DOI:** 10.1371/journal.pone.0334934

**Published:** 2025-10-21

**Authors:** Rodrigo Riera, Joana Vasconcelos, Susanne Baden, Alejandro Martínez, Eduardo Infantes

**Affiliations:** 1 BIOCON, IU-ECOAQUA, Universidad de Las Palmas de Gran Canaria, Las Palmas de Gran Canaria, Canary Islands, Spain; 2 2MARE - Marine and Environmental Sciences Centre/ARNET - Aquatic Research Network, Agência Regional para o Desenvolvimento da Investigação Tecnologia e Inovação (ARDITI), Funchal, PORTUGAL; 3 Department of Biological and Environmental Sciences, University of Gothenburg, Kristineberg, Fiskebäckskil, Sweden; 4 Molecular Ecology Group (MEG), Water Research Institute (IRSA), National Research Council of Italy (CNR), Verbania, Italy; King Abdulaziz University, SAUDI ARABIA

## Abstract

Seagrasses are highly sensitive to human-induced disturbances and global environmental changes. Since the 1980s, *Zostera marina* meadows along the West Swedish coast (Skagerrak) have declined significantly, as evidenced by changes in morpho-anatomical traits, reductions in area coverage, and shifts in associated communities. However, infaunal assemblages within *Z. marina* meadows remain understudied compared to epifaunal communities and have not been previously used as indicators of seagrass regression. To investigate spatial variability in infaunal composition, we analysed samples from 15 coastal stations at depths of 1.5–3 m depth. Using an n-dimensional hypervolume framework, we assessed functional differences between infaunal and epifaunal communities. We examined infaunal community descriptors—such as species richness and individual abundance—biotic indices, environmental drivers (including wave exposure and *Z. marina* biomass), and correlations with epifauna. Variability in infaunal composition across sampling stations was primarily driven by differences in the abundance of dominant taxa, including the polychaete *Capitella capitata*, oligochaetes, nematodes, and chironomids. Several coastal stations, such as Marstrand and Finsbo, were classified as moderately polluted, though biotic indices, i.e., AMBI, M-AMBI and ISI, showed discrepancies. Spatial patterns in infaunal assemblages were mainly influenced by *Z. marina* biomass and maximum fetch, with a good representation of oligochaetes and chironomids in exposed stations. These findings suggest that infauna respond differently from epifauna but provide valuable additional insights into the ecological status, functional traits, and trophic diversity of *Z. marina* meadows. Integrating multiple community components is essential for a more comprehensive understanding of the processes and patterns driving seagrass ecosystem regression.

## Introduction

Human-driven disturbances are widespread across coastal areas worldwide [1,2], and their extent and variety have increased significantly in recent decades. In addition to long-established stressors such as eutrophication and chemical contamination, recently recognised or increasingly relevant pressures, such as microplastics, underwater noise, and light pollution, are intensifying their impacts on marine ecosystems [3–5]. Minimizing and mitigating these disturbances is an urgent priority at global, national, and regional levels [6]. A precise understanding of their ecological impacts is essential for advancing knowledge of coastal dynamics and processes [7]. Consequently, effective spatial management planning must consider not only the total surface area of ecosystems but also a comprehensive understanding of the biological structures and environmental drivers shaping them [8]. A thorough grasp of biodiversity responses to human-induced pressures is fundamental to ensuring that human activities remain within planetary boundaries and to accurately assessing their environmental impact in the Anthropocene [9].

Ecological baselines—reference states of habitat distribution, species composition, and abundance—are essential for informing conservation and management strategies [10]. Biodiversity monitoring programs provide crucial data on ecosystem changes, guiding research, conservation assessments, and future planning [11]. However, despite their importance, biodiversity data remain scarce and fragmented, with substantial geographic and taxonomic gaps, compounded by inconsistencies in sampling methodologies. These challenges are more pronounced in marine environments, where data collection requires extensive logistical resources, including specialized equipment, boat access, and substantial financial investment [12].

Biodiversity monitoring is particularly effective for assessing the environmental quality of marine habitats [13]. Polluted sites typically exhibit shifts in species composition, favouring taxa that thrive under altered environmental conditions. Several biotic indices have been developed to quantify ecological status based on benthic community structure. These include the Multivariate-AZTI Marine Biotic Index (M-AMBI), the Benthic Quality Index (BQI), Norwegian Sensitivity Index (NSI), Norwegian Quality Index 1 (NQI1), the Infaunal Trophic Index (ITI) and Infaunal Sensitivity Index (ISI) [14]. Most of these tools are based on the Pearson-Rosenberg model [15] which describes community responses to organic enrichment and disturbance along environmental gradients. These indices were developed to assess the ecological quality of soft-bottom benthic habitats, focusing primarily on subtidal infaunal communities [16]. As a result, they often overlook valuable ecological information provided by other components of the benthic assemblage, such as epifauna and demersal fish.

Prioritizing the monitoring of highly impacted environments is crucial for conservation and coastal management. Among marine ecosystems, seagrass meadows have been a focal point of conservation efforts due to their extensive global decline [17]. The loss of seagrass meadows extends beyond the disappearance of *Zostera marina* itself; it also disrupts associated communities of infauna, epifauna, and fish, leading to a sharp decline in coastal biodiversity [18,19]. Despite the recognized importance of ecological baselines for coastal management, integrative studies incorporating multiple seagrass-associated communities remain scarce (though see [8] for an exception). This is paradoxical given the well-established role of benthic invertebrates in bentho-pelagic coupling. These organisms play a fundamental role in coastal ecosystem processes, including nutrient cycling, detritus decomposition, and carbon transfer to higher trophic levels [20–23]. Incorporating multiple faunal communities into seagrass monitoring programs could enhance our understanding of ecosystem dynamics and the effects of anthropogenic disturbances, particularly when considering long-lived species with varying mobility, such as infauna and epifauna [24].

Infauna and epifauna differ significantly in trophic composition (e.g., infauna are predominantly detritivores, whereas epifauna are often herbivores), life history traits (e.g., prey-predator interactions), and niche utilization. These differences could provide deeper insights into ecological patterns and processes occurring within seagrass meadows [8]. More importantly, their contrasting responses to disturbances—due to differences in mobility—may offer additional perspectives on ecosystem changes. While infaunal species generally have limited mobility and directly exposed to environmental stressors, epifauna, with their greater mobility, can potentially evade localized disturbances within the same meadow [25]. However, these distinctions have yet to be explicitly tested.

This study aims to investigate the potential contributions of infaunal communities in *Zostera marina* meadows to biodiversity monitoring and environmental assessments. We focus on infaunal communities along the Skagerrak coast (Swedish west coast), spanning approximately through a coastline distance of 200 km [26]. Our main objective is structured into five specific aims: (i) to characterise the infaunal communities in terms of taxonomic composition, abundance, species richness, and functional traits; (ii) to assess the ecological health status of the sampled seagrass meadows using established biotic indices (M-AMBI, ISI, NSI, NQI1, ITI, and BENTIX); (iii) to explore the factors influencing the taxonomic and functional composition of infaunal assemblages across sampling stations, considering both *Zostera*-related variables (e.g., *Z. marina* biomass and meadow surface area) and environmental factors (e.g., hydrodynamic exposure); and (iv) to determine the relationship between infaunal and epifaunal assemblages across coastal sites, and (v) evaluate whether epifauna can serve as a proxy for long-term eutrophication effects in the region [26,27]. By integrating taxonomic and functional dimensions of biodiversity with environmental context, this study contributes to a more holistic understanding of how benthic assemblages respond to multiple pressures in temperate seagrass ecosystems. This multifaceted perspective helps interpret patterns in community structure and ecosystem functioning, while also mitigating some limitations inherent to single-season sampling.

## Materials and methods

### Study area

**Fig 1:**
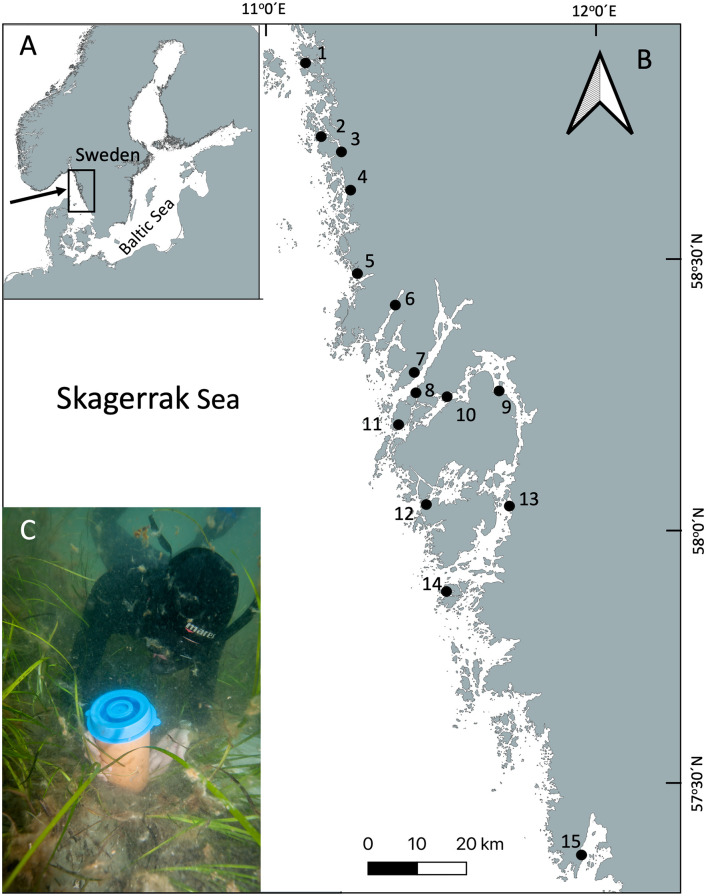
Study areas along the Swedish West Coast. **(a)** Locations of the 15 sampled seagrass meadows in Sweden. **(b)** Detailed view of the study sites along the Swedish West Coast: 1. **N.** Lindholmen; 2. Kvarnekilen; 3. **S.** Stridsfjorden (Sannäsfjorden); 4. Kämpersvik; 5. Valön; 6. Bottnefjord; 7. Finsbo; 8. Lindholmen; 9. Slussen; 10. Hjältön; 11. Skalhavet; 12. Björnholmen; 13. Kåkenäs; 14. Marstrand; 15. Gottskärsviken. **(c)** Infauna sampling in a seagrass meadow using a sediment core (QGIS, v3.2, https://qgis.org/).

A total of 15 *Zostera marina* meadows were sampled along the Swedish Skagerrak coast in July 2018 (Fig 1, Table 1), coinciding with the peak abundance and biomass of epibenthic invertebrates [28]. These meadows were located in shallow waters (1–3 m depth) and spanned approximately 200 km of coastline, encompassing a range of hydrodynamic conditions.

**Table 1:** Mean abundance of infauna and leaf epifauna in *Zostera marina* meadows.

Nº	Meadow name	Latitude N	Longitude E	Infauna > 0.5 mm (ind.m^-2^)	Epifauna > 1 mm (ind.m^-2^)	Epifauna > 0.25 mm (ind.m^-2^)	Max.Fetch (km)	Wave exposure
				(Present work)	(Riera et al. [26])			
**1**	**N. Lindholmen**	58°53’20.0	11°8’2.2	393 ± 84	859 ± 317	35975 ± 8851	0.5	Sheltered
**2**	**Kvarnekilen**	58°44’58.2	11°11’3.9	314 ± 90	4226 ± 1534	73023 ± 11724	0.8	Sheltered
**3**	**S. Stridsfjorden**	58°43’12.4	11°15’16.1	471 ± 91	7396 ± 1777	134237 ± 31784	2.7	Semi-exposed
**4**	**Kämpersvik**	58°38’51.6	11°17’2.4	2414 ± 721	270 ± 77	12220 ± 1961	2.3	Semi-sheltered
**5**	**Valön**	58°29′24.4	11°18′7.9	903 ± 239	0*	0*	1.6	Semi-sheltered
**6**	**Bottnefjord**	58°27’51.3	11°19’4.7	883 ± 184	1252 ± 359	38504 ± 17179	5.2	Exposed
**7**	**Finsbo**	58°18’6.2	11°29’33.2	1610 ± 154	971 ± 172	51179 ± 5663	3.5	Semi-exposed
**8**	**Lindholmen**	58°15’46.5	11°29’48.7	923 ± 283	36029 ± 10543	80027 ± 12339	0.5	Sheltered
**9**	**Slussen**	58°15’45.9	11°47’5.6	903 ± 157	939 ± 347	24866 ± 3736	8.1	Exposed
**10**	**Hjältön**	58°15’16.6	11°36’15.5	1295 ± 334	885 ± 170	30630 ± 4360	4.5	Semi-exposed
**11**	**Skalhavet**	58°12’13.8	11°26’3.7	2885 ± 468	3429 ± 1826	72784 ± 17674	1.3	Sheltered
**12**	**Björnholmen**	58°3’8.2	11°31’26.3	687 ± 193	10275 ± 6248	89056 ± 36536	3.4	Semi-exposed
**13**	**Kåkenäs**	58°2’44.6	11°48’35.9	1963 ± 473	268 ± 86	14917 ± 2882	15.1	Exposed
**14**	**Marstrand**	57°53’14.9	11°35’10.0	2257 ± 715	22091 ± 7583	116319 ± 19595	1.4	Sheltered
**15**	**Gottskärsviken**	57°23’4.8	12°1’18.4	177 ± 40	3576 ± 1443	41228 ± 9231	6.9	Exposed

The mean abundance (ind.m^-2^ ± SE) of infauna (> 0.5 mm) and leaf epifauna (> 1 mm and > 0.25 mm) in 15 *Z, marina* meadows along the Swedish west coast in July 2018. * No *Zostera* meadows were reported during the field survey. Epifauna data extracted from Riera et al. [26].

To evaluate the influence of hydrodynamics on eelgrass fauna composition, we quantified wave exposure using the maximum fetch for each site (Table 1). Maximum fetch is defined as the longest horizontal distance over which wind-driven waves can develop and is considered the most reliable predictor of sediment composition. This is because storms originating from non-dominant wind directions can also affect sediment properties. Given that wave exposure influences sediment grain size, organic content, and nutrient availability in eelgrass sediments [29], we selected meadows along a gradient of wave exposures. For clarity, we categorized these into four exposure levels based on maximum fetch values: sheltered (0.5–1.5 km), semi-sheltered (1.9–2.4 km), semi-exposed (5.1–6.9 km), and exposed (8.7–13.3 km) [30]. However, data on sediment composition were not collected from the sampling sites.

### Field sampling and sample processing

Infaunal samples were randomly collected using a PVC core (Ø10 cm, length 30 cm) at the selected stations. At each meadow, cores were inserted to a depth of 20 cm in vegetated habitats and immediately transferred to plastic bags. A total of six cores were taken per location, with each replicate spaced at least 2 m apart to minimize the bow-wave effect. Samples were collected by snorkelling. In the laboratory, the samples were sieved through a 0.5 mm mesh, after which the infauna was collected, preserved in 70% ethanol, and stained with Bengal rose. Specimens were identified to species level whenever possible and counted using a stereomicroscope.

For epifaunal samples, six randomly selected eelgrass specimens with associated fouling (detritus, epiphytes, and fauna) were collected per meadow using a plankton net with a 200 µm (0.2 mm) mesh size, mounted on a frame enclosing an area of 35 × 35 cm (0.123 m²). Each sample was spaced at least 5 m apart and was collected by snorkelling. The coverage of eelgrass and macroalgae was estimated in the field using a 50 × 50 cm frame placed within the meadow, with replicates spaced at least 5 m apart.

In the laboratory, *Z. marina* leaves were rinsed with freshwater to remove detritus, and the associated epiphytes and fauna were sieved through a 1 mm (1,000 µm) mesh (“large epifauna”) and a 200 µm (0.2 mm) mesh (“small epifauna”). Although both fractions of epifauna occupy the lowest trophic levels, they are considered distinct assemblages due to previously reported differences in species composition [28,31,32]. Therefore, they were analysed separately in this study (see Riera et al. [26] for further details on epifauna sampling and *Zostera* biomass).

All plant material was collected in accordance with national and international guidelines and legislation, with efforts made to minimize disturbance to the sampled meadows.

### Functional space characterization

Our functional analyses followed the general protocol proposed by Mammola et al. [33], beginning with the creation of a trait matrix based on input data from the associated communities of *Z. marina* meadows. This matrix was then used to delineate the multidimensional trait space to describe patterns and responses to ecological questions. In this analysis, we aimed to assess whether functional space differed between infauna and small and large epifauna. We expected to observe greater polarization in functional trait distributions among these communities, potentially influenced by wave exposure.

To explore and visualize the functional differences between infaunal and epifaunal communities in *Z. marina* meadows, we constructed a functional trait matrix for each of the 70 recorded taxa using published literature (Table 2). This matrix was then used to represent the functional space of each community through a geometric n-dimensional hypervolume approach [34,35]. The functional characteristics of epifaunal and infaunal communities were compared to assess the extent of overlap between them, indicating whether they fulfil similar roles in ecosystem functioning. This probabilistic method employs high-dimensional kernel density estimations to delineate the shape and volume of the multidimensional space [35].

**Table 2:** Biological trait variables and categories for functional matrix construction.

Traits	Categories
**Maximum body size**	mm
**Trophic guild**	Omnivorous, Subsurface depositivorous, Detritivorous, Carnivorous, Suspensivorous, Grazer
**Mobility**	Crawler, Burrower, Semi motile
**Elongated body**	Present, Absent
**Larvae development**	Direct, Indirect
**Eyes**	Present, Absent

Six biological traits were selected for their relevance in distinguishing among the three studied faunal communities—namely, infauna, large epifauna, and small epifauna (Table 2, S1 Table) [36,37]. These traits represent key aspects of organism morphology (“maximum body size,” “presence of elongated body,” and “presence of eyes”), feeding strategies (“trophic guild”), behavior (“mobility”), and life history characteristics (“larval development”). Each trait reflects a specific dimension of ecological functioning in benthic communities: maximum body size is related to resource use and susceptibility to predation [36]; trophic guild captures the species’ role in energy flow (e.g., detritivory vs. suspension feeding) [36,38]; mobility influences sediment reworking and exposure to environmental stressors [37]; the presence of eyes is associated with predator avoidance and active foraging [37,39]; an elongated body facilitates burrowing and sediment penetration [38]; and larval development mode relates to dispersal capacity and recovery potential following disturbance [38]. When information on a specific trait was unavailable for a given taxon, zero values were assigned for each trait category, and the taxon was excluded from trait weighting calculations. The selected functional traits are commonly used in invertebrate studies [36–39].

Trait categories were defined for each of the 70 recorded taxa in infauna, large epifauna, and small epifauna communities in the studied *Zostera marina* meadows (S1 Table). Trait selection and classification were based on commonly used references in trait-based benthic studies [36–39], and specific trait sources are cited below. Definitions of each trait and their ecological relevance are provided in the main text.

As our dataset consisted predominantly of categorical traits, with only “maximum body size” as a continuous variable, we used the Gower dissimilarity measure to accommodate mixed data types and performed Principal Component Analysis (PCA) to extract orthogonal morphological axes [40]. Hypervolumes were then calculated using the package BAT v. 2.7.0 [41] in the software R [42], implementing a Gaussian kernel density estimate. The Gaussian kernel density estimation was selected because it allows for a probabilistic, rather than binary, characterization of functional space [40], a method that has been successfully applied to capture functional variation in similar marine environments [43].

To determine whether infaunal and epifaunal communities across meadows with different exposure levels were subjected to distinct filtering processes, we quantified the dispersion of functional space using the *kernel.dispersion()* function and the divergence method [40].

### Statistical analysis

Univariate metrics, including abundance, species richness (number of taxa), Shannon–Wiener Index (H’ [44]), Hurlbert Index (ES50 [45]), Pielou’s Evenness (J’ [46]), AMBI (AZTI’s Marine Biotic Index [16]), Norwegian Sensitivity Index (NSI [47]), Indicator Species Index (ISI [47]), and BENTIX [48], were calculated for infauna, large epifauna, and small epifauna. Additionally, as multimetric or multivariate methods, M-AMBI (multivariate AMBI [16,49]) and BQI (Benthic Quality Index [13,50]) were applied. With the exception of BQI (calculated as average abundances per station), all indices were computed at the replicate level. AMBI and M-AMBI were calculated using AMBI index software v5.0 (http://ambi.azti.es), while NSI, ISI, and BENTIX were computed using the *BBI* R package v. 0.3.0 [51]. The remaining metrics were calculated using the *vegan* R package v. 2.6–6.1 [52]. The BQI formulation used in the Swedish assessment within the Water Framework Directive (WFD) was applied in this study [13].

Boundary settings for the BQI classification followed Rosenberg et al. [50] in accordance with WFD recommendations, where coastal environmental status is categorized into five levels. In the present study, BQI values ranged from 1.70 to 7.12 (reference value) for coastal stations at depths <20 m, corresponding to the five WFD ecological status classes. The classification was as follows: BQI ≥ 5.70: “High” ecological status; BQI ≥ 4.2: “Good” ecological status; BQI ≥ 2.85: “Moderate” ecological status; BQI ≥ 1.42: “Poor” ecological status; BQI < 1.42: “Bad” ecological status.

To ensure statistical independence and avoid pseudoreplication, replicate cores were first averaged within each site (n = 15 meadows). All subsequent multivariate and regression-based analyses were therefore conducted at the site level, with one set of response variables (infaunal composition and diversity indices) per meadow.

The analysis minimised overfitting from collinearity and ensured model stability by calculating site-level pairwise Spearman’s rank correlations among all candidate environmental predictors using the *corrplot* R package v.0.95 [53]. Where two predictors were stronlgy correlated (R² > 0.6 [54]), only the biologically most relevant predictor [55] was retained. This screening yielded eight site-level predictors: *Zostera marina* biomass, *Chorda filum* biomass, *Fucus serratus* biomass, *Dictyota* sp. Biomass, *Polysiphonia* sp. Biomass, *Cladophora* sp. Biomass, *Aglaothamnion* sp. Biomass, and Maximum fetch.

To assess patterns in infaunal assemblage structure among the 15 *Zostera marina* meadows, non-metric multidimensional scaling (n-MDS) was conducted in three dimensions (k = 3), based on the Bray-Curtis dissimilarities of relative abundances. Abundance data were standardised by row totals after adding a small constant, without transformation. Ordinations were performed using the *vegan* R package v2.6.6. [52]. Species potentially driving site distribution patterns were identified using the *envfit* function in *vegan* [52], retaining taxa with significant correlations (p ≤ 0.013).

Distance-based redundancy analysis (db-RDA) and multivariate generalised linear models (GLMs) were applied, using the averaged site-level community matrix (n = 15) as the response data and eight predictor variables as covariates. To test whether environmental variation (e.g., *Zostera marina* biomass, eelgrass coverage, and associated algal biomasses) structured infaunal assemblages across the 15 *Z. marina* meadows, we performed a db-RDA [56] with the *dbrda()* function from the vegan R package [52], computing Bray–Curtis dissimilarities on square-root-transformed, site-level relative abundances. Predictor collinearity was screened using Variance Inflation Factors (VIFs), adopting a threshold of <10. Model and axes significance were tested by permutation (999 permutations) with *anova.cca()* function. Ordination was visualised with a biplot showing sites grouped by wave exposure category (sheltered, semi-sheltered, semi-exposed, exposed), and environmental vectors indicating the direction and strength of association.

Complementary inference used GLMs fitted to the site-level infaunal abundance matrix with *manyglm()* function (*mvabund* R package v.4.2.1) [57], specifying a negative binomial distribution to accommodate overdispersion. Candidate predictors were selected a priori based on the db-RDA results. Significance (deviance tests and p-values) was evaluated with *anova.manyglm()* function using PIT-trap resampling (999 iterations). Model assumptions were evaluated by visual inspection of residual plots and quantile-quantile (QQ) plots [54]. This approach provided a robust, model-based test of the multivariate effect of environmental predictors on infaunal community composition.

Correlations between univariate variables (e.g., individual abundance, number of species, H’, J’, and ES50) for infaunal and epifaunal communities, as well as environmental variables (*Z. marina* and epiphyte biomass), were tested using the Pearson product-moment correlation when both variables followed a normal distribution. When normality was not met, the Spearman rank correlation was applied to assess the degree of association between variables.

The evenness of trait distributions within the total functional space was assessed using the *kernel.evenness* function of *BAT* R package v.2.9.6 [32], which quantifies the overlap between the observed functional hypervolume and a theoretical hypervolume with evenly distributed traits and abundances. The significance of observed differences in functional richness, dispersion, and evenness was tested using a Wilcoxon test.

## Results

### Infauna composition

A total of 18,078 individuals were collected, representing 37 taxa across eight taxonomic groups: Polychaetes, Mollusks, Oligochaetes, Insects, Amphipods, Echinoderms, Nemerteans, and Nematodes (S2 Table). The most abundant taxa included the polychaete *Capitella capitata* (2,885 ind.m ⁻ ², 16% of total abundance), followed by oligochaetes (2,650 ind.m ⁻ ², 15%) and chironomids (2,454 ind.m ⁻ ², 14%). In contrast, nine taxa—*Aonides oxycephala*, *Magelona* sp., *Nephtys* sp., *Phyllodoce maculata*, *Sphaerosyllis histryx*, *Spionidae* sp. 1, the mollusk *Macoma balthica*, the echinoderm *Asterias rubens*, and Nemerteans—were recorded in only a single replicate, indicating their rarity.

**Fig 2:**
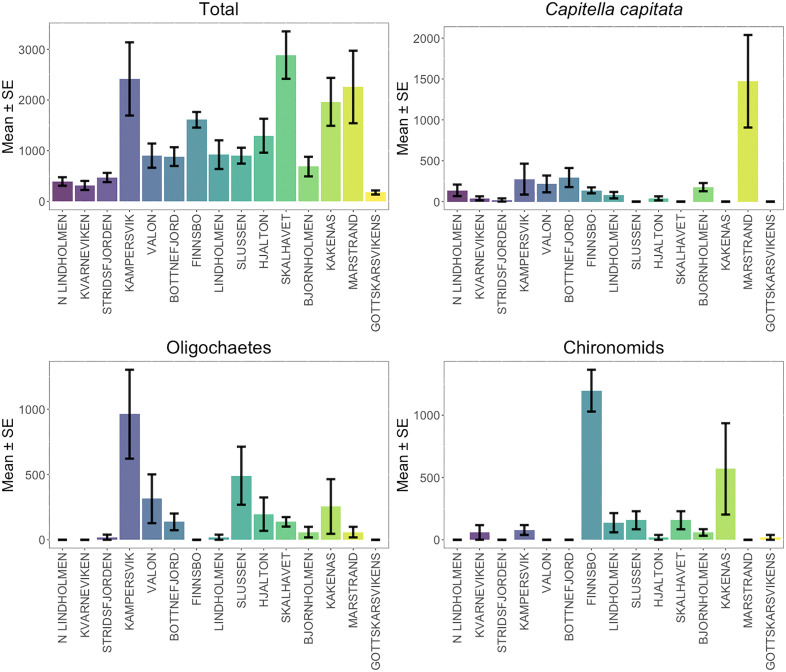
Mean abundances of dominant infauna species. Mean abundances (ind.m^-2^ ± SE) of the most abundant infauna species in each of the 15 *Zostera marina* meadows sampled in 2018 along the Swedish west coast.

Species richness varied among sampling stations, with the highest diversity recorded at Kämpersvik (17 species) and Skalhavet (15 species). The lowest richness was observed at Gottskärsvikens and Kvarneviken (6 species each) (S2 Table). Mean abundance also fluctuated across stations, with Kämpersvik, Skalhavet, and Marstrand exhibiting the highest densities (>2,000 ind.m ⁻ ²), while Gottskärsvikens (177 ind.m ⁻ ²) and Kvarneviken (314 ind.m ⁻ ²) had the lowest (Fig 2, Table 1).

**Fig 3:**
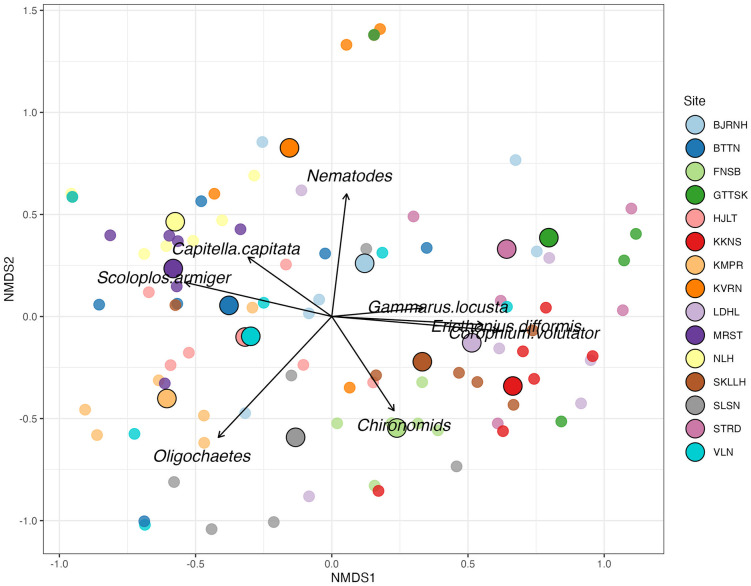
n-MDS of infaunal assemblages in *Zostera marina* meadows. Non-metric multidimensional scaling (n-MDS) of replicate samples (n = 88) from 15 *Z. marina* meadows, based on Bray–Curtis dissimilarity of relative abundances. Points are coloured by site; larger circles with black outlines represent site-level centroids. Vectors indicate taxa most strongly correlated with the ordination (envfit, p ≤ 0.013) (Stress: 0.19; 3D solution). BJRNH, Björnholmen; BTTN, Bottnefjord; FNSB, Finsbo; GTTSK, Gottskärsviken; HJLT, Hjältön; KKNS, Kåkenäs; KMPR, Kämpersvik; LDHL, Lindholmen; MRST, Marstrand; NLH, N. Lindholmen; SKLH, Skalhavet; SLSN, Slussen; STRD, S. Stridsfjorden; VLN, Valön.

The n-MDS ordination plot (Fig 3) revealed no clear clustering of infaunal composition across meadows, although some site centroids were positioned farther from the ordination centre, reflecting community divergence. Importantly, Finsbo (FNSB) was distinguised by a high chironomids abundance, Lindholmen (LDHL) by elevated densities of *Corophium volutator* and *Ericthonius difformis*, Marstrand (MRST) by *Scoloplos armiger,* North Lindholmen (NLH) by *Capitella capitata* and Kvarnekilen (KKNS) by the highest nematode abundances (S2 Table). Overall, infaunal composition exhibited high spatial variability among meadows, driven primarily by differences in the relative abundances of Oligochaetes, *Corophium volutator*, Nematodes, *Scoloplos armiger, Erichtonius difformis,* Chironomids, *Capitella capitata*, and *Gammarus locusta* (Fig 3).

**Fig 4:**
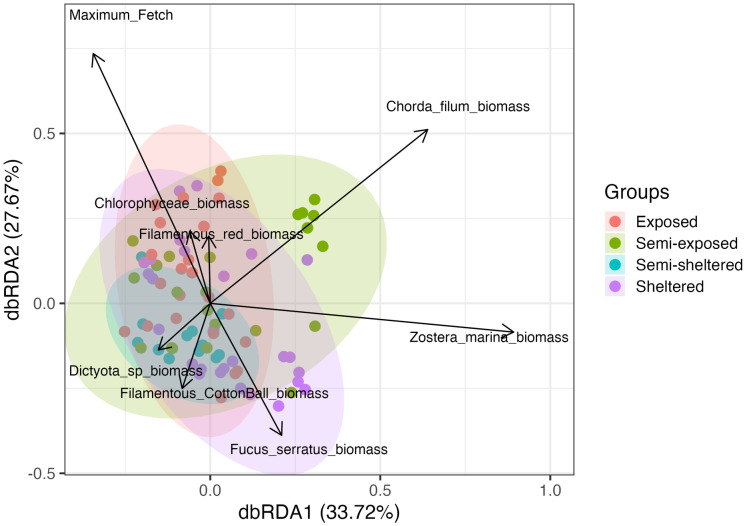
Distance-based redundancy analysis (db-RDA) ordination plot of infaunal assemblages. The ellipses represent 95% confidence intervals around replicate cores grouped by wave exposure category (sheltered, intermediate, exposed). Black arrows represent the most influential environmental variables structuring infaunal communities across *Zostera marina* meadows. Only the first two canonical axes are shown.

Db-RDA showed that maximum fetch, *Z. marina* biomass, and *Chorda filum* biomass were the most influential predictors of infaunal assemblage variation across sites. The first two canonical axes explained 61.4% of the total variation (Axis 1 = 33.7%, Axis 2 = 27.7%) (Fig 4).

Comparisons of nested Generalized Linear Models (GLMs) using analysis of deviance showed consistent relationships between environmental predictors and infaunal composition, with Akaike Information Criterion (AIC) supporting the same pattern. Both models achieved comparable fit across the 15 *Z. marina* meadows. Model 1 (*Z. marina* biomass, *Chorda filum* biomass, *Fucus serratus* biomass, and maximum fetch), identified *Z. marina* biomass (p = 0.036) and maximum fetch (p = 0.042) as significant predictors. Model 2 (Model 1 plus biomass of *Polysiphonia* sp., *Cladophora* sp., *Aglaothamnion* sp., and *Dictyota* sp.), produced similar results: *Z. marina* biomass remained significant (p = 0.039), and maximum fetch was marginally significant (p = 0.080). In both models, the remaining algal biomasses contributed little or no explanatory power. In both models, the remaining algal biomasses contributed little additional explanatory power. Overall, the GLMs consistently highlighted *Z. marina* biomass and maximum fetch as the primary predictors of infaunal community composition (Table 3).

**Table 3:** Multivariate Generalized Linear Model (GLM) results testing the relationship between infaunal assemblage structure and environmental variables in *Zostera marina* meadows.

	Res. Df	Df. diff	Dev	Pr(<Dev)
**Model 1**				
**(Intercept)**	89			
**Infauna x *Zostera marina* biomass**	88	1	20.20	0.036**
**Infauna x *Chorda filum* biomass**	87	1	19.14	0.243
**Infauna x Maximum fetch**	85	1	52.26	0.042**
**Model 2**				
**Infauna x *Zostera marina* biomass**	88	1	20.20	0.039**
**Infauna x *Chorda filum* biomass**	87	1	19.14	0.225
**Infauna x *Fucus serratus* biomass**	86	1	13.07	0.620
**Infauna x Maximum fetch**	85	1	53.90	0.080*
**Infauna x *Filamentous CottonBall* biomass**	84	1	28.37	0.342
**Infauna x Chlorophyceae biomass**	83	1	46.41	0.265
**Infauna x *Filamentous_*red biomass**	82	1	13.05	0.644
**Infauna x *Dictyota* sp.**	81	1	9.42	0.606

Model 1 includes three variables identified as the most influential from the db-RDA biplot. Model 2 includes all available predictors. Significance levels: p < 0.05 **; p < 0.1 *.

### Biotic indices

Contrasting results were obtained in the ecological status assessment of the sampling meadows. AMBI values presented the most optimistic scenario, varying widely across meadows, from 1.17 (“undisturbed”) in Gottskärsviken to 4.49 (“moderately disturbed”) in Marstrand (Table 4). The mean AMBI value was 3.15, corresponding to a “slightly disturbed” ecological status. With the exception of Gottskärsviken (“undisturbed”), all stations fell into either the “slightly disturbed” or “moderately disturbed” categories.

**Table 4:** Ecological indices and quality status of infaunal communities.

Nº	Meadow name	N	S	H’	AMBI	M-AMBI	ISI	NSI	BQI	BENTIX
**1**	**N. Lindholmen**	393 (236–707)	8	1.77	3.82	0.59	4.71	13.47	3.11	2.00
**2**	**Kvarnekilen**	314 (118–589)	6	1.44	3.42	0.51	7.57	19.03	1.71	2.00
**3**	**S. Stridsfjorden**	471 (236–707)	11	1.96	2.28	0.76	4.45	16.51	5.79	2.29
**4**	**Kämpersvik**	2414 (707–5300)	17	2.03	3.54	0.84	5.65	16.12	5.15	2.51
**5**	**Valön**	903 (118–1767)	11	1.95	4.18	0.65	5.66	12.00	4.44	2.0
**6**	**Bottnefjord**	883 (353–1413)	9	1.74	4.32	0.58	4.74	13.07	3.93	2.00
**7**	**Finsbo**	1610 (1295–2238)	7	0.97	3.45	0.48	4.21	13.27	1.70	2.21
**8**	**Lindholmen**	923 (353–2238)	11	1.87	1.94	0.77	1.58	6.98	7.12	2.00
**9**	**Slussen**	903 (707–1531)	9	1.50	3.89	0.56	6.53	21.27	4.57	2.00
**10**	**Hjältön**	1295 (707–2709)	12	1.70	3.17	0.69	5.24	18.37	5.13	2.00
**11**	**Skalhavet**	2885 (1178–4240)	15	2.25	1.98	0.91	7.30	20.04	6.67	2.193
**12**	**Björnholmen**	687 (118–1413)	10	2.11	3.19	0.72	1.58	6.98	3.37	2.00
**13**	**Kåkenäs**	1963 (1178–4240)	9	1.83	2.36	0.70	NaN	NaN	4.70	2.00
**14**	**Marstrand**	2257 (471–4946)	10	1.24	4.49	0.57	6.86	9.37	2.46	2.00
**15**	**Gottskärsviken**	177 (118–353)	6	1.74	1.17	0.70	NaN	NaN	4.83	2.00

Total abundance (N, mean values per m^2^ with total range in brackets), species richness (S), Shannon diversity index (H’), and benthic biotic indices with their ecological quality status: AZTI’s Marine Biotic Index (AMBI), Norwegian Sensitivity Index (NSI), Indicator Species Index (ISI), BENTIX and M-AMBI for the analysed datasets. The ecological quality status for AMBI follows this color scheme: Undisturbed = Blue; Slightly disturbed = Green; and Moderately disturbed = Yellow. For M-AMBI, ISI and NSI indexes, the ecological quality status is represented as Blue = Very Good; Green = Good; Yellow = Moderate; Orange = Bad; and Red = Very Bad. The coloration applied in BQI follows WFD recommendations; Red = Bad, Orange = Poor, Yellow = Moderate, Green = Good, and Blue = High ecological status.

Due to a low number of species in Gottskärsviken and the dominance of chironomids and oligochaetes in Kåkenäs, NSI and ISI indices were not assigned to these stations (S2 Table). In contrast, the BENTIX index exhibited little variation, ranging from 2 to 2.51 (Table 4).

M-AMBI varied between 0.48 (Finsbo) and 0.91 (Skalhavet), with a mean of 0.69 (“moderately disturbed”). Three meadows were classified as “undisturbed”: Lindholmen (0.77), Kämpersvik (0.84), and Skalhavet (0.91). Two meadows were categorized as “moderately disturbed”: Finsbo (0.48) and Kvarnekilen (0.51). The remaining sampling stations were identified as “slightly disturbed”, with values ranging from 0.56 to 0.76 (Table 4).

BQI varied greatly across the sampled *Z. marina* meadows, with a mean value of 4.31, corresponding to a “Good” ecological status. The lowest BQI values were recorded in Kvarnekilen (1.71, “Poor”) and Finsbo (1.70, “Poor”), whereas the highest were found in Lindholmen (7.12, “High ecological status”) and Skalhavet (6.67, “High ecological status”) (Table 4).

### Comparative functional approach between infauna, epifauna, and environmental variables

**Fig 5:**
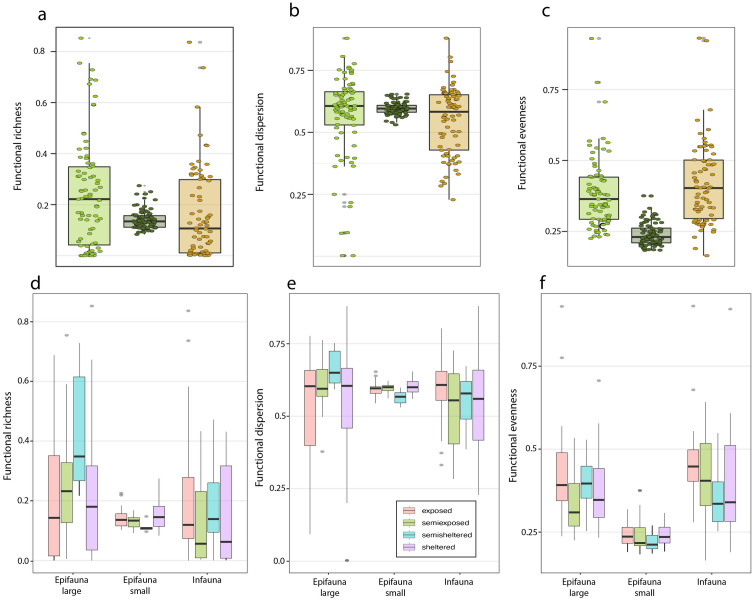
Functional richness, dispersion, and evenness across large and small epifauna, and infaunal communities and wave exposure levels. Overall differences in (a) functional richness, (b) functional dispersion, and (c) functional evenness between habitats, as well as across meadows with varying degrees of wave exposure **(d–f)**. Epifauna data extracted from Riera et al. [26].

Analyses used the first four principal components, which together explained 91.4% of the variance, with the default per-axis bandwidth. Twelve replicates with one or no species were excluded. From the remaining data, 237 hypervolumes were reconstructed. The functional space showed only slight polarization (S1 Fig.); nevertheless, functional diversity, dispersion, and evenness differed significantly among assemblages^‌^. Specifically, functional diversity was significantly higher in large epifauna compared to infauna (W = 3531, p = 0.026) and between small and large epifauna (W = 4112, p < 0.05). Functional evenness was significantly lower in small epifauna compared to both large epifauna (W = 6067, p < 0.001) and infauna (W = 532, p < 0.001) (Fig 5f). Marginally significant differences were also found between large and small epifauna (W = 4103, p = 0.009) and between small epifauna and infauna (W = 3608, p = 0.082) (Fig 5a–5c). Within each community, no clear trends were observed regarding the degree of wave exposure across meadows (Fig 5d–5e).

Comparison of infaunal and epifaunal communities based on univariate descriptors (Shannon diversity, Pielou’s evenness, Hurlbert index, individual abundance, and species richness) showed no consistent patterns (Table 5). Additionally, correlations between infaunal descriptors and biomass of *Z. marina* and associated algae revealed no clear trends, except for two significant relationships, (i) Species richness *vs. Z. marina* biomass and (ii) Species richness *vs. Polysiphonia* sp. biomass (Table 5).

**Table 5:** Correlation between infaunal parameters, epifauna, and environmental variables.

Variable	Infauna vs. Large Epifauna	Infauna vs. Small Epifauna	Infauna vs. *Zostera marina* biomass	Infauna vs. *Chorda filum* biomass	Infauna vs. *Polysiphonia* sp. biomass	Infauna vs. *Fucus serratus* biomass
**Abundance**	−0.196	−0.113	−0.324	0.069	0.312	−0.144
**Richness (S)**	−0.091	0	−0.463*	0.139	0.571**	−0.328
**Shannon–Wiener Index (H’)**	0.087	−0.027	–	–	–	–
**Pielou’s Evenness (J’)**	0.165	−0.284	–	–	–	–
**Hurlbert index (ES50)**	−0.105	−0.024	–	–	–	–

Correlation coefficients relating univariate parameters of infauna with epifauna and environmental variables. Significance levels: p < 0.001 ***; p < 0.05 **; p < 0.1 *. Epifauna data extracted from Riera et al. [26].

## Discussion

### Infaunal assemblages dynamics and environmental drivers

Infaunal assemblages varied among meadows in the study area, primarily due to shifts in the most abundant taxa, namely the capitellid polychaete *Capitella capitata*, *Ericthonius difformis*, *Scoloplos armiger*, oligochaetes, nematodes, and chironomids. In contrast, non-dominant taxa played a minor role in differentiating infaunal assemblages among the sampled stations. Although *Zostera marina* meadows are known to provide stable and favourable conditions for infaunal communities – such as sediment stability, reduced water movement, and increased sedimentation facilitated by their rhizomes and roots [58] – the studied meadows supported a low-diversity infaunal assemblage. Our site-level analysis highlights that variation in *Z. marina* biomass and Maximum Fetch are the primary environmental drivers structuring infaunal communities.

Studies on infauna from Scandinavian *Z. marina* meadows are limited and heterogeneous in terms of methodologies and spatio-temporal sampling efforts [19,59–61]. Collectively, these studies indicate that infaunal abundance in *Z. marina* meadows is three times higher than in adjacent non-vegetated sediments [59,61]. This difference may be attributed to variations in grain size and organic content (e.g., Dahl et al. [28]; Baden & Pihl [30,60]) as well as the increased habitat complexity provided by rhizomes.

Wave exposure can influence meadow distribution [62] and reduce seagrass survival through erosion [63,64], which in turn affects sediment grain size and habitat suitability for benthic invertebrates. Grain size is largely governed by flow velocities, particularly by the threshold of critical shear stress required to mobilise sediment particles [65,66]. Species richness within infaunal communities often increases with coarser sediments due to enhanced pore-water flow [67,68] due to enhanced pore-water flow, as observed in exposed sampling stations. Although we lacked direct grain size measurements or finding that Maximum Fetch significantly predicts community composition suggests that hydrodynamic forcing plays a key role.

Seagrass canopies attenuate wave energy by slowing the water flow beneath the leaves, promoting fine-sediment deposition and creating stable microhabitats for benthic organisms, including infauna and larvae settlement [69–72]. In our study, wave exposure – quantified by Maximum Fetch- emerged as one of the most influential environmental drivers shaping infaunal community composition, alongside with *Zostera marina* biomass. However, species richness did not vary consistently with exposure level: the highest value was recorded at a semi-sheltered meadow (Kämpersvik), while both sheltered (Kvarnekilen) and exposed (Gottskärsviken) meadows exhibited the lowest richness (Table 5). Other univariate descriptors, such as individual abundance and evenness, also showed no clear patterns across the exposure gradient.

Historical data are available for Norra Lindholmen (1999), Finsbo (1980–82, 1999), and Lindholmen (1982, 1999) [19,60]. Infaunal abundance at these stations exhibited significant spatial and temporal variability. However, a marked 15-fold decline has been reported when comparing data from the 1980s and 1990s to the present study. A similar downward trend was observed in epifaunal assemblages of *Z. marina* in the study area, largely driven by declines in small amphipods and Mytilus edulis plantigrades [19,25,60,73]. Infaunal changes over the past four decades appear closely linked to declining coastal environmental quality.

### Biotic indices and constraints in ecological assessment

Ecological status was assesses using six biotic indices: AMBI, M-AMBI, BENTIX, ISI, NSI, BQI. These revealed a wide range of conditions across stations, from “Bad” to “Very Good.”. ISI, NSI, and BENTIX displayed the greatest variability among meadows, while AMBI and M-AMBI yielded more intermediate scores. Most *Zostera marina* meadows were neither heavily disturbed nor fully undisturbed. Some sampling stations (e.g., Marstrand and Finsbo) were consistently classified as moderately disturbed, reflecting high abundances of the opportunistic polychaete *Capitella capitata* (Marstrand) and chironomid larvae (Finsbo). These taxa are widely recognised as indicators of degraded water and sediment conditions [74,75] and contributed to reduced richness and evenness. Continued inter-calibration of biotic indices across water body types remains essential for ensuring consistent and reliable ecological assessments [76]. At Marstrand, long-term decline in eelgrass cover has been attributed to sediment resuspension and associated turbidity [26,77], while Finsbo proximity to a busy ferry route may contribute to ongoing environmental stress.

Caution is warranted when interpreting biotic index results, as several stations exhibited very low species richness, while others were dominated by taxa identified only to higher taxonomic levels- factor that affected the performance of indices such as BENTIX, ISI, and NSI. Additionally, a substantial proportion of taxa could not be assigned to specific ecological groups. The indices applied in this study have traditionally been used in unvegetated subtidal sediments at depths greater than 5 m and to our knowledge, this represents their first application in shallow *Zostera marina* meadows (1–5 m depth). Although not originally designed for intertidal or shallow habitats, recent studies have shown promising outcomes when applied to broad intertidal zones with tidal ranges exceeding >2 m (e.g., [78]). However, shallow subtidal environments (1–5 m) remain poorly represented in ecological assessments compared to deeper sandy seabeds, including those within fjords, coastal lagoons, and estuaries (see [13,78]). In this study, sampling was conducted in *Z. marina* meadows at depths between 1.5 and 3 m. Early work by Gislen [79] documented eelgrass beds ranging from 0.9 to 8.4 m, whereas the current mean maximum depth is approximately 4 m [26,80], reflecting a notable reduction in *Z. marina* habitat along the Swedish Skagerrak coast since 1926.

### Epifauna-infauna interactions, functional traits and long-term trends

Changes in the composition and proliferation of epiphytic algae on *Z. marina* leaves may indirectly influence infaunal assemblages, given the trophic and functional interdependence between epifaunal and infaunal communities. However, no significant correlations were found between infaunal and epifaunal assemblages at the sampled meadows, suggesting that these groups respond differently to variation in *Z. marina* meadow structure. One possible explanation lies in the influence of seagrass roots and rhizomes on sedimentary conditions, including reduced sediment erosion [72] and microbial-driven processes such as nutrient cycling and organic matter decomposition [81]. Notably, belowground biomass production in many seagrasses can equal that of leaf tissue [82]. Several studies have highlighted the importance of buried seagrass structures in shaping infaunal communities. Although some degree of epifauna-infauna interdependence is likely, epifaunal assemblages appear to respond more directly to aboveground features, with denser *Z. marina* meadows typically supporting higher epifaunal diversity and abundance than sparse ones [26].

Functional hypervolume analysis of large and small epifauna, as well as infauna, revealed limited differentiation among these communities. Nevertheless, some functional metrics differed significantly across assemblages. These findings indicate that both epifauna and infauna communities contribute similarly to the overall functional space of *Z. marina* meadows, with no distinct segregation in trait composition. Their contributions, however, vary in quantitative terms. Large epifauna and infauna were predominantly composed of species with intermediate densities, whereas small epifauna were dominated by two to three amphipod species, resulting in lower functional diversity and evenness. A relatively high number of species were shared between epifaunal and infaunal habitats. Although typically recorded at low abundances, these species contributed to the homogenization of functional space, particularly when traits were assessed at the species level rather than the individual level. To better resolve potential functional differences, future studies should incorporate measurements of morphological traits, particularly body size, at the individual level within each environment. This could reveal stronger functional contrasts, as *Z. marina* leaves and sediments may serve as distinct microhabitats for different developmental stages of a given species. Similar ontogenetic habitat shifts have been documented in meiofauna within *Posidonia oceanica* meadows, where dominant mite species display clear life-stage-specific habitat preferences d [42,43].

From a temporal perspective, epifaunal assemblages exhibited clear trends when compared to historical data, reflecting broad regional shifts in *Z. marina*-associated fauna over the past two decades [26]. In contrast, the paucity of historical data on infaunal communities limits our ability to assess long-term changes in these assemblages. The regression of *Z. marina* meadows over recent decades appears to affect not only the seagrass itself—an important ecosystem engineer—but also associated biota, including epifauna, infauna, and fish. While epifaunal trends were relatively coherent across meadows, infaunal communities showed high spatial variability, suggesting site-specific differences among meadows. Riera et al. [26,83] demonstrated that large and small epifauna serve as effective proxies for detecting changes in *Z. marina* meadows in Skagerrak region. In contrast, infaunal patterns were more heterogeneous, and no consistent correlations emerged between epifaunal and infaunal communities. This aligns with findings by Reiss et al. [8], who reported significant correlations between the two groups only in terms of species richness.

### Integrative ecological insights and management implications

This integrative analysis identified *Zostera* marina biomass and Maximum Fetch as key drivers of infaunal community structure along the Skagerrak coast. These factors influenced infaunal composition independently of biotic indices used to assess ecosystem condition and appeared more influential than the presence of other associated faunal groups, such as epifauna. The structural complexity provided by seagrass rhizomes and their stabilising role in exposed environments underscore the ecological importance of *Z. marina* meadows. Infaunal assemblages offer valuable insights into sediment quality and are sensitive to multiple stressors, including detritus accumulation, hydrodynamic conditions, and contamination. In parallel, the composition of epiphytic algae plays a critical role in shaping epifaunal communities, highlighting the need to consider both above- and belowground processes when assessing the integrity of seagrass ecosystems.

Infaunal communities are widely used as bioindicators of ecological condition, as shifts in their composition often reflect increasing levels of environment disturbance [15]. Given their ecological relevance, *Z. marina* meadows and their associated infaunal assemblages should be integrated into coastal management strategies, particularly in restoration planning and efforts to mitigate environmental stressors. The pronounced spatial variability in infauna composition observed across the study area also suggests potential differences in underlying ecosystem processes, such as secondary production and fine-scale sedimentary dynamics, which merit further investigation.

The integrative framework applied in this study may also be extended to other coastal ecosystems, including salt marshes, estuarine and lagoon environments. These habitats support diverse infaunal and epifaunal communities that, when assessed together, offer a more holistic view of ecosystem functioning. Such an approach addresses important gaps in ecological evaluation that arise when studies focus on a single biological component—such as epifauna—while overlooking other communities and key environmental drivers like maximum fetch.

## Conclusions

This study highlights the complex interplay between physical drivers, habitat structure, and benthic community composition in *Zostera marina* meadows along the Skagerrak coast. Infaunal assemblages showed marked spatial variability, primarily driven by variation in *Z. marina* biomass and hydrodynamic exposure (Maximum Fetch). These factors shaped community composition regardless of biotic indices or associated faunal groups, reinforcing the need to consider structural habitat attributes and local environmental forcing in benthic assessments.

Although *Z. marina* meadows are typically associated with enhanced biodiversity, the observed low infaunal diversity and the dominance of opportunistic taxa in some locations indicate potential environmental stress. Biotic indices provided useful, though occasionally inconsistent, insights into ecological condition due to limitations in taxonomic resolution and the application of indices developed for deeper, unvegetated sediments.

Functional trait analysis revealed that both epifaunal and infaunal communities contribute similarly to the overall ecological functioning of seagrass meadows, despite compositional differences. Their combined assessment provides a more complete picture of benthic ecosystem integrity, especially when informed by traits at the individual level.

Long-term data revealed significant declines in infaunal abundance over recent decades, mirroring known reductions in *Z. marina* coverage and environmental quality. This emphasizes the importance of long-term monitoring and habitat conservation. The integrative framework applied here—linking physical habitat characteristics with taxonomic and functional metrics of multiple faunal groups—offers a promising approach for ecological assessment and coastal management. Its application to other shallow coastal habitats could improve our ability to detect and respond to environmental change at ecologically meaningful scales.

## Supporting information

S1 Table Trait assignment matrix for infaunal and epifaunal taxa [see 36–39 for details]. (DOCX)

S2 Table Mean abundance (ind m^-2^ bottom) of infauna species associated to *Zostera marina* meadows, per each of the 15 stations sampled along the Swedish west coast in July 2018. 1 – N. Lindholmen; 2 – Kvarnekilen; 3 – S. Stridsfjorden (Sannäsfjorden); 4 – Kämpersvik; 5 – Valön; 6 – Bottnefjord; 7 – Finsbo; 8 – Lindholmen; 9 – Slussen; 10 – Hjältön; 11 – Skallhavet; 12 – Björnholmen; 13 – Kåkenäs; 14 – Marstrand; 15 – Gottskärsviken.
(DOCX)

S1 Fig Functional space of 237 macrobenthic communities associated with *Zostera marina*, visualised in a 4-dimensional hypervolume. Communities include large epifauna (light green), small epifauna (dark green), and infauna (brown). Each hypervolume is represented by 1,000 random points, coloured according to community identity. Larger points represent the centroids of each hypervolume. Due to the proximity of centroids, many appear visually superimposed.
(TIF)
