# Supplementary material for: Towards a more integrative environmental assessment: Infauna as tool for Zostera marina conservation management
Source: PLoS One. 2025 Oct 21;20(10):e0334934. doi: 10.1371/journal.pone.0334934 (PMC12539717; doi:10.1371/journal.pone.0334934)
Supplement: S1 Table — (DOCX) [file pone.0334934.s001.docx]

1. **Supplementary Tables and Figures**
   1. **Tables**

**S1 Table.** Trait assignment matrix for infaunal and epifaunal taxa [see 36-39 for details].

| **Species** | **Maximum body size (mm)** | **Trophic guild** | **Mobility** | **Elongated body** | **Larval development** | **Eyes** |
| --- | --- | --- | --- | --- | --- | --- |
| **INFAUNA** | | | | | | |
| **Polychaeta** | | | | | | |
| Aonides oxycephala | 100 | Carnivorous | Burrower | Y | Indirect | Y |
| Capitella capitata | 120 | Subsurface depositivorous | Burrower | Y | Indirect | N |
| *Capitellidae* sp1 | 150 | Subsurface depositivorous | Burrower | Y | Indirect | N |
| *Capitella* sp. | 100 | Subsurface depositivorous | Burrower | Y | Indirect | N |
| *Eteone longa* | 50 | Carnivorous | Burrower | Y | Indirect | Y |
| *Harmothoe* sp | 25 | Carnivorous | Crawler | Y | Indirect | Y |
| *Magelona* sp | 170 | Surface depositivorous | Burrower | Y | Indirect | Y |
| *Malacoceros fuliginosus* | 114 | Carnivorous | Burrower | Y | Indirect | Y |
| *Nephtys* sp. | 200 | Carnivorous | Crawler | Y | Indirect | N |
| *Nephtys hombergii* | 200 | Carnivorous | Crawler | Y | Indirect | N |
| *Hediste diversicolor* | 200 | Carnivorous | Crawler | Y | Indirect | Y |
| *Alitta virens* | 900 | Carnivorous | Crawler | Y | Indirect | Y |
| *Phyllodoce maculata* | 100 | Carnivorous | Burrower | Y | Indirect | Y |
| *Pseudopolydora antennata* | 30 | Carnivorous | Burrower | Y | Indirect | Y |
| *Scoloplos armiger* | 120 | Subsurface depositivorous | Burrower | Y | Indirect | N |
| *Sphaerosyllis histryx* | 5 | Omnivorous | Burrower | Y | Indirect | Y |
| Spionidae sp1 | 30 | Carnivorous | Burrower | Y | Indirect | Y |
| **Mollusca** | | | | | | |
| Parvicardium pinnulatum | 13 | Suspensivorous | Semimotile | N | Indirect | N |
| Hiatella arctica | 40 | Suspensivorous | Semimotile | N | Indirect | N |
| Macoma balthica | 25 | Suspensivorous | Semimotile | N | Indirect | N |
| Musculus discors | 13 | Suspensivorous | Semimotile | N | Indirect | N |
| Mya arenaria | 150 | Suspensivorous | Semimotile | N | Indirect | N |
| Nassarius sp. | 12 | Grazer | Burrower | N | Indirect | N |
| Rissoa sp. | 10 | Grazer | Burrower | N | Indirect | N |
| **Amphipoda** | | | | | | |
| *Corophium volutator* | 10 | Subsurface depositivorous | Crawler | N | Direct | Y |
| *Ericthonius brasiliensis* | 10 | Omnivorous | Crawler | N | Direct | Y |
| *Ericthonius difformis* | 10 | Omnivorous | Crawler | N | Direct | Y |
| *Gammarus* sp. | 20 | Omnivorous | Crawler | N | Direct | Y |
| *Gammarus locusta* | 33 | Omnivorous | Crawler | N | Direct | Y |
| *Maera* sp. | 15 | Omnivorous | Crawler | N | Direct | Y |
| *Microdeutopus gryllotalpa* | 10 | Omnivorous | Crawler | N | Direct | Y |
| **Malacostraca** | | | | | | |
| *Palaemon adspersus* | 70 | Omnivorous | Swimmer | Y | Indirect | Y |
| **Chironomids (Insecta)** | | | | | | |
| varia | 15 | Omnivorous | Crawler | Y | Indirect | Y |
| **Echinodermata** | | | | | | |
| *Asterias rubens* | 520 | Carnivorous | Burrower | N | Indirect | N |
| **Nematodes** | | | | | | |
| varia | 10 | Omnivorous | Burrower | Y | Direct | N |
| **Nemertea** | | | | | | |
| varia | 30 | Carnivorous | Burrower | Y | Indirect | N |
| **Oligochaeta** | | | | | | |
| varia | 20 | Detritivorous | Burrower | Y | Indirect | N |
| **LARGE EPIFAUNA** | | | | | | |
| **Polychaeta** | | | | | | |
| Capitellidae sp1 | 150 | Subsurface depositivorous | Burrower | Y | Indirect | N |
| *Lepidonotus squamatus* | 50 | Carnivorous | Crawler | Y | Indirect | Y |
| *Platynereis dumerilii* | 50 | Omnivorous | Crawler | Y | Indirect | Y |
| *Neanthes aff. virens* | 110 | Omnivorous | Crawler | Y | Indirect | Y |
| Maldanidae sp1 | 100 | Subsurface depositivorous | Burrower | Y | Indirect | N |
| *Prionospio* sp. | 50 | Omnivorous | Burrower | Y | Indirect | Y |
| *Harmothoe* sp. | 25 | Carnivorous | Crawler | Y | Indirect | Y |
| **Mollusca** | | | | | | |
| *Cerastoderma edule* | 560 | Suspensivorous | Semimotile | N | Indirect | N |
| *Chiton* sp. | 50 | Grazer | Burrower | Y | Indirect | N |
| *Musculus discors* | 13 | Suspensivorous | Semimotile | N | Indirect | N |
| *Mya arenaria* | 100 | Suspensivorous | Semimotile | N | Indirect | N |
| *Nassarius nitidus* | 20 | Grazer | Burrower | N | Indirect | Y |
| *Nudibranchia* sp1 | 150 | Grazer | Burrower | N | Indirect | Y |
| *Rissoa aff. lilacina* | 50 | Grazer | Burrower | N | Indirect | Y |
| *Rissoa* sp. | 50 | Grazer | Burrower | N | Indirect | Y |
| Mitilids | 13 | Suspensivorous | Semimotile | N | Indirect | N |
| *Gibbula cinearia* | 16 | Grazer | Burrower | N | Indirect | Y |
| *Littorina littorea* | 30 | Grazer | Burrower | N | Indirect | Y |
| *Aeolidiella glauca* | 45 | Grazer | Burrower | N | Indirect | Y |
| **Crustacea** | | | | | | |
| *Paleamon adspersus* | 70 | Omnivorous | Swimmer | Y | Indirect | Y |
| *Idotea baltica* | 30 | Omnivorous | Crawler | Y | Direct | Y |
| *Carcinus maenas* | 86 | Carnivorous | Crawler | N | Direct | Y |
| *Macropodia rostrata* | 23 | Carnivorous | Crawler | N | Direct | Y |
| *Crangon crangon* | 69 | Carnivorous | Crawler | N | Direct | Y |
| *Inarchus dorsettensis* | 50 | Carnivorous | Crawler | N | Direct | Y |
| *Pagurus benhardus* | 80 | Omnivorous | Burrower | Y | Direct | Y |
| **Chironomids (Insecta)** | | | | | | |
| Chironomid larvae | 15 | Omnivorous | Crawler | Y | Indirect | Y |
| **Echinodermata** | | | | | | |
| *Asterias rubens* | 520 | Carnivorous | Burrower | N | Indirect | N |
| *Psammechinus miliaris* | 57,5 | Omnivorous | Burrower | N | Indirect | N |
| *Echinus esculentus* | 176 | Omnivorous | Burrower | N | Indirect | N |
| **Oligochaeta** | | | | | | |
| Enchytraeidae sp1 | 20 | Detritivorous | Burrower | Y | Indirect | N |
| **Nemertea** | | | | | | |
| Nemertea sp1 | 30 | Carnivorous | Burrower | Y | Indirect | N |
| **Urochordata** | | | | | | |
| *Ciona intestinalis* | 200 | Suspensivorous | Sessile | N | Indirect | N |
| **Cnidaria** | | | | | | |
| *Sagartiogeton viduatus* | 25 | Suspensivorous | Semimotile | N | Indirect | N |
| Cnidaria sp1 | 30 | Suspensivorous | Semimotile | N | Indirect | N |
| **Fishes** | | | | | | |
| *Gobius niger* | 180 | Carnivorous | Swimmer | Y | Indirect | Y |
| *Syngnathus typhle* | 250 | Carnivorous | Swimmer | Y | Indirect | Y |
| **SMALL EPIFAUNA** | | | | | | |
| **Polychaeta** | | | | | | |
| *Nereis* sp. fragments | 50 | Carnivorous | Crawler | Y | Indirect | Y |
| Polychaetes varia | 50 | Carnivorous | Crawler | Y | Indirect | Y |
| **Mollusca** | | | | | | |
| Mitilids | 100 | Suspensivorous | Semimotile | N | Indirect | N |
| *Rissoa* spp. | 15 | Grazer | Burrower | N | Indirect | Y |
| *Littorina littorea* | 30 | Grazer | Burrower | N | Indirect | Y |
| *Pecten maximus* | 60 | Suspensivorous | Semimotile | N | Indirect | N |
| *Cardium* sp. | 50 | Suspensivorous | Semimotile | N | Indirect | N |
| Mollusca varia | 50 | Suspensivorous | Semimotile | N | Indirect | N |
| **Amphipoda** | | | | | | |
| *Erichtonius difformis* | 10 | Omnivorous | Crawler | N | Direct | Y |
| *Monocorophium insidiosum* | 10 | Subsurface depositivorous | Crawler | N | Direct | Y |
| *Microdeutopus gryllotalba* | 10 | Omnivorous | Crawler | N | Direct | Y |
| *Caprella* sp. | 25 | Carnivorous | Crawler | Y | Direct | Y |
| *Gammarus* sp. | 20 | Omnivorous | Crawler | N | Direct | Y |
| **Isopoda** | | | | | | |
| *Idotea* sp. | 30 | Omnivorous | Crawler | Y | Direct | Y |
| **Copepoda** | | | | | | |
| Harpacticoids | 10 | Detritivorous | Burrower | Y | Indirect | Y |
| **Crustacea** | | | | | | |
| Crustacea varia | 50 | Carnivorous | Crawler | N | Direct | Y |
| **Insecta** | | | | | | |
| Chironomids | 15 | Carnivorous | Crawler | Y | Indirect | Y |
| Fly larvae | 15 | Carnivorous | Crawler | Y | Indirect | Y |
| **Chelicerata** | | | | | | |
| *Thalassarachna basteri* | 10 | Carnivorous | Burrower | N | Indirect | Y |
| **Echinodermata** | | | | | | |
| *Asterias rubens* | 520 | Carnivorous | Burrower | N | Indirect | N |
| *Echinus esculentus* | 176 | Grazer | Burrower | N | Indirect | N |
| **Oligochaeta** | | | | | | |
| *Enchytraeidae* sp. | 20 | Detritivorous | Burrower | Y | Indirect | N |
| *Tubificidae* sp. | 20 | Detritivorous | Burrower | Y | Indirect | N |
| **Nemertea** | | | | | | |
| *Nemertea* sp. | 30 | Carnivorous | Burrower | Y | Indirect | N |
| **Nematoda** | | | | | | |
| *Southernia zosterae* | 15 | Omnivorous | Burrower | Y | Indirect | Y |
| **Sipuncula** | | | | | | |
| *Sipunculidae* sp. | 100 | Detritivorous | Burrower | Y | Indirect | N |
| **Plathelmyntha** | | | | | | |
| *Turbellaria* sp. | 50 | Carnivorous | Crawler | Y | Indirect | Y |
| **Phoraminiferans** | | | | | | |
| Phoraminiferans | 10 | Detritivorous | Semimotile | N | Direct | N |
| **Acari** | | | | | | |
| *Acari* sp. | 10 | Carnivorous | Burrower | N | Indirect | Y |
