# Supplementary material for: Towards a more integrative environmental assessment: Infauna as tool for Zostera marina conservation management
Source: PLoS One. 2025 Oct 21;20(10):e0334934. doi: 10.1371/journal.pone.0334934 (PMC12539717; doi:10.1371/journal.pone.0334934)
Supplement: S2 Table — 1 – N. Lindholmen; 2 – Kvarnekilen; 3 – S. Stridsfjorden (Sannäsfjorden); 4 – Kämpersvik; 5 – Valön; 6 – Bottnefjord; 7 – Finsbo; 8 – Lindholmen; 9 – Slussen; 10 – Hjältön; 11 – Skallhavet; 12 – Björnholmen; 13 – Kåkenäs; 14 – Marstrand; 15 – Gottskärsviken. (DOCX) [file pone.0334934.s002.docx]

1. **Supplementary Tables and Figures**

**S2 Table.** Mean abundance (ind m^-2^ bottom) of infauna species associated to *Zostera marina* meadows, per each of the 15 stations sampled along the Swedish west coast in July 2018. 1 - N. Lindholmen; 2 – Kvarnekilen; 3 - S. Stridsfjorden (Sannäsfjorden); 4 – Kämpersvik; 5 – Valön; 6 – Bottnefjord; 7 – Finsbo; 8 – Lindholmen; 9 – Slussen; 10 – Hjältön; 11 – Skallhavet; 12 – Björnholmen; 13 – Kåkenäs; 14 – Marstrand; 15 – Gottskärsviken.

| **Species** | **1** | **2** | **3** | **4** | **5** | **6** | **8** | **9** | **10** | **11** | **12** | **13** | **14** | **15** | **18** |
| --- | --- | --- | --- | --- | --- | --- | --- | --- | --- | --- | --- | --- | --- | --- | --- |
| **Crustacea** | | | | | | | | | | | | | | | |
| *Palaemon adspersus* | 0 | 0 | 0 | 39 | 0 | 0 | 0 | 20 | 0 | 0 | 0 | 0 | 0 | 0 | 0 |
| *Corophium volutator* | 0 | 0 | 216 | 0 | 79 | 79 | 79 | 137 | 20 | 20 | 530 | 20 | 471 | 20 | 20 |
| *Erichtonius brasiliensis* | 0 | 0 | 0 | 0 | 0 | 0 | 0 | 20 | 0 | 0 | 0 | 0 | 0 | 0 | 20 |
| *Erichtonius difformis* | 0 | 0 | 39 | 0 | 59 | 20 | 39 | 353 | 59 | 39 | 707 | 39 | 177 | 0 | 39 |
| *Gammarus* sp. | 0 | 0 | 0 | 20 | 0 | 0 | 0 | 0 | 0 | 0 | 157 | 0 | 0 | 0 | 0 |
| *Gammarus locusta* | 0 | 0 | 20 | 0 | 39 | 20 | 0 | 98 | 20 | 0 | 236 | 118 | 294 | 0 | 0 |
| *Maera sp* | 0 | 0 | 0 | 0 | 0 | 0 | 0 | 0 | 0 | 0 | 0 | 0 | 59 | 0 | 0 |
| *Microdeutopus gryllotalpa* | 0 | 0 | 0 | 0 | 0 | 0 | 0 | 20 | 0 | 0 | 0 | 0 | 59 | 0 | 0 |
| **Mollusca** | | | | | | | | | | | | | | | |
| *Parvicardium pinnulatum* | 0 | 0 | 0 | 39 | 0 | 0 | 0 | 0 | 0 | 0 | 98 | 0 | 0 | 0 | 0 |
| *Hiatella arctica* | 0 | 0 | 20 | 118 | 0 | 0 | 0 | 0 | 0 | 0 | 59 | 0 | 0 | 0 | 0 |
| *Macoma balthica* | 0 | 0 | 0 | 0 | 0 | 0 | 0 | 0 | 0 | 0 | 20 | 0 | 0 | 0 | 0 |
| *Musculus discors* | 0 | 0 | 20 | 0 | 0 | 0 | 0 | 0 | 0 | 0 | 20 | 0 | 0 | 0 | 0 |
| *Mya arenaria* | 0 | 0 | 39 | 255 | 39 | 20 | 0 | 0 | 0 | 20 | 79 | 0 | 0 | 59 | 0 |
| *Nassarius* sp. | 20 | 0 | 20 | 0 | 0 | 0 | 0 | 0 | 20 | 20 | 0 | 79 | 20 | 0 | 0 |
| *Rissoa* sp. | 0 | 0 | 0 | 0 | 0 | 0 | 0 | 0 | 0 | 0 | 0 | 0 | 0 | 0 | 39 |
| **Echinodermata** | | | | | | | | | | | | | | | |
| *Asterias rubens* | 0 | 0 | 0 | 0 | 0 | 0 | 0 | 0 | 0 | 20 | 0 | 0 | 0 | 0 | 0 |
| **Nematodes** | | | | | | | | | | | | | | | |
| Varia | 0 | 157 | 39 | 20 | 20 | 0 | 0 | 0 | 0 | 0 | 0 | 59 | 0 | 0 | 39 |
| **Nemertea** | | | | | | | | | | | | | | | |
| Varia | 0 | 0 | 0 | 0 | 0 | 0 | 0 | 0 | 0 | 0 | 20 | 0 | 0 | 0 | 0 |
| **Oligochaeta** |  |  |  |  |  |  |  |  |  |  |  |  |  |  |  |
| Varia | 0 | 0 | 20 | 962 | 314 | 137 | 0 | 20 | 491 | 196 | 137 | 59 | 255 | 59 | 0 |
| **Polychaeta** | | | | | | | | | | | | | | | |
| *Aonides oxycephala* | 0 | 0 | 0 | 0 | 0 | 0 | 20 | 0 | 0 | 0 | 0 | 0 | 0 | 0 | 0 |
| Capitellidae sp1 | 0 | 0 | 0 | 39 | 0 | 0 | 0 | 0 | 0 | 0 | 0 | 20 | 0 | 0 | 0 |
| *Capitella* sp. | 0 | 0 | 0 | 0 | 39 | 0 | 0 | 0 | 0 | 0 | 0 | 0 | 0 | 0 | 0 |
| *Capitella capitata* | 137 | 39 | 20 | 275 | 216 | 294 | 137 | 79 | 0 | 39 | 0 | 177 | 0 | 1472 | 0 |
| *Eteone longa* | 20 | 0 | 0 | 20 | 0 | 0 | 20 | 0 | 0 | 59 | 0 | 0 | 0 | 20 | 0 |
| *Harmothoe* sp. | 0 | 20 | 0 | 0 | 39 | 0 | 0 | 0 | 0 | 20 | 0 | 0 | 0 | 0 | 0 |
| *Magelona* sp. | 0 | 0 | 0 | 0 | 0 | 0 | 0 | 0 | 0 | 0 | 0 | 0 | 0 | 20 | 0 |
| *Malacoceros fuliginosus* | 20 | 0 | 0 | 20 | 0 | 0 | 0 | 0 | 0 | 0 | 0 | 0 | 0 | 79 | 0 |
| *Nephtys* sp. | 0 | 0 | 0 | 0 | 0 | 0 | 0 | 0 | 20 | 0 | 0 | 0 | 0 | 0 | 0 |
| *Nephtys hombergii* | 0 | 0 | 0 | 20 | 0 | 0 | 0 | 0 | 0 | 0 | 0 | 0 | 0 | 20 | 0 |
| *Hediste diversicolor* | 39 | 0 | 20 | 177 | 39 | 59 | 0 | 20 | 59 | 589 | 393 | 59 | 59 | 255 | 0 |
| *Alitta virens* | 0 | 0 | 0 | 20 | 0 | 0 | 0 | 20 | 0 | 0 | 39 | 0 | 0 | 0 | 0 |
| *Phyllodoce maculata* | 20 | 0 | 0 | 0 | 0 | 0 | 0 | 0 | 0 | 0 | 0 | 0 | 0 | 0 | 0 |
| *Pseudopolydora antennata* | 39 | 0 | 0 | 0 | 0 | 20 | 0 | 0 | 0 | 0 | 0 | 0 | 0 | 0 | 0 |
| *Scoloplos armiger* | 98 | 20 | 0 | 294 | 20 | 236 | 118 | 0 | 59 | 255 | 236 | 0 | 0 | 255 | 0 |
| *Sphaerosyllis hystrix* | 0 | 20 | 0 | 0 | 0 | 0 | 0 | 0 | 0 | 0 | 0 | 0 | 0 | 0 | 0 |
| Spionidae sp1 | 0 | 0 | 0 | 20 | 0 | 0 | 0 | 0 | 0 | 0 | 0 | 0 | 0 | 0 | 0 |
| **Insecta** | | | | | | | | | | | | | | | |
| Chironomids | 0 | 59 | 0 | 79 | 0 | 0 | 1197 | 137 | 157 | 20 | 157 | 59 | 569 | 0 | 20 |
