# Supplementary figures and images for: Towards a more integrative environmental assessment: Infauna as tool for Zostera marina conservation management
Source: PLoS One. 2025 Oct 21;20(10):e0334934. doi: 10.1371/journal.pone.0334934 (PMC12539717; doi:10.1371/journal.pone.0334934)

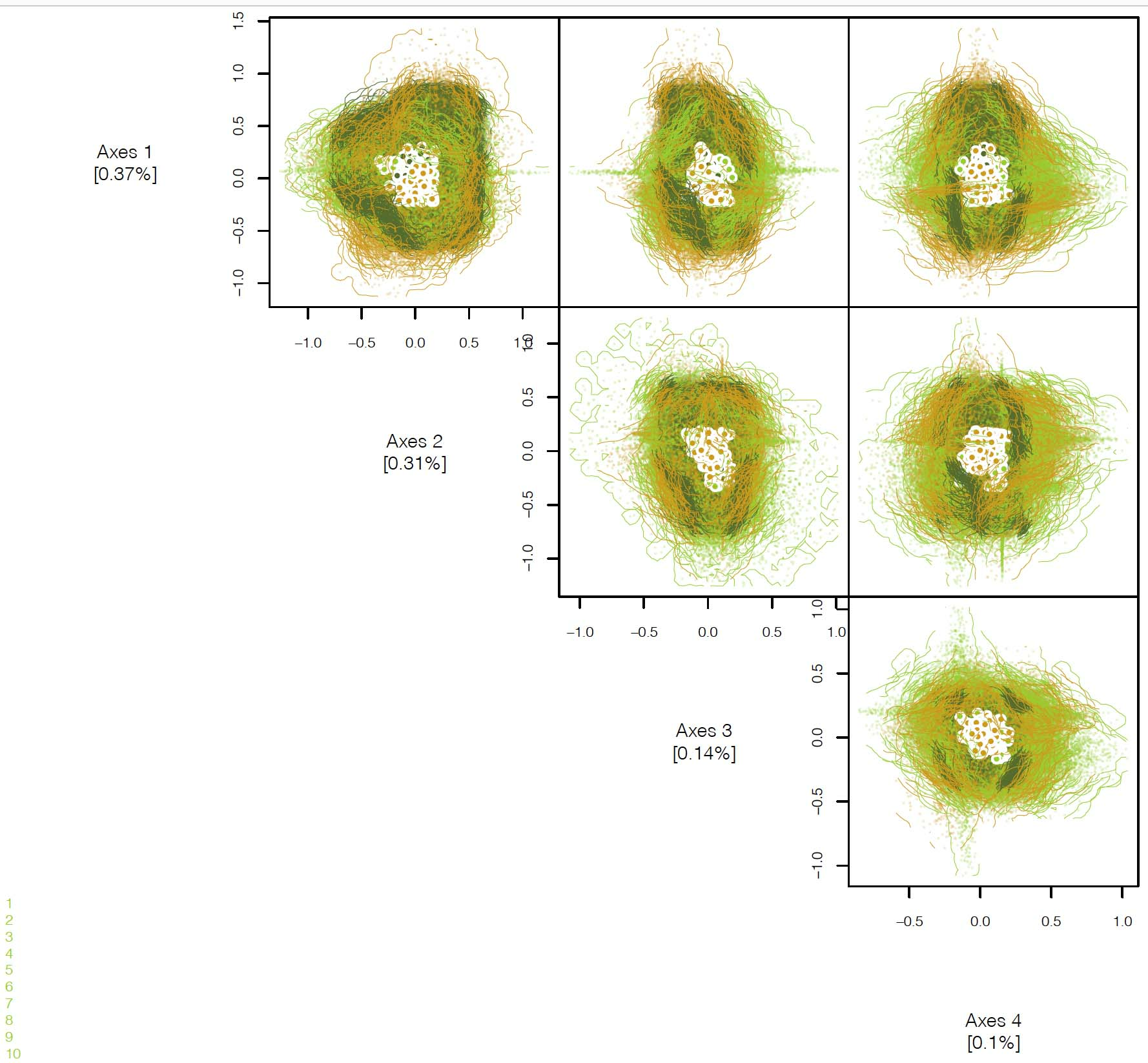

Supplement: S1 Fig — Communities include large epifauna (light green), small epifauna (dark green), and infauna (brown). Each hypervolume is represented by 1,000 random points, coloured according to community identity. Larger points represent the centroids of each hypervolume. Due to the proximity of centroids, many appear visually superimposed. (TIF) [file pone.0334934.s003.tif]
